# A Rare Case of T-cell Lymphoblastic Lymphoma: A Diagnostic Predicament

**DOI:** 10.7759/cureus.65994

**Published:** 2024-08-02

**Authors:** Malabika Shil, Vaishnavi Srinivasan, Anjali A Vincent, Trupti Gaikwad, Delphia Thomas

**Affiliations:** 1 Oral Medicine and Radiology, Mithila Minority Dental College and Hospital, Darbhanga, IND; 2 Oral Medicine, Government Dental College and Hospital, Hyderabad, IND; 3 Oral and Maxillofacial Pathology, Government Dental College and Hospital, Hyderabad, IND; 4 Oral Medicine and Radiology, Government Dental College and Hospital, Hyderabad, IND; 5 Oral Medicine and Radiology, Dr. D. Y. Patil Dental College and Hospital, Dr. D. Y. Patil Vidyapeeth, Pune, IND; 6 Oral Medicine and Radiology, All India Institute of Medical Sciences, Raipur, Raipur, IND

**Keywords:** non-hodgkin’s lymphoma, oral lymphoma, lymphoblast, t cell, t-cell lymphoma

## Abstract

Lymphomas are the malignant neoplasms of lymphocytes and their precursor cells. Their diagnosis can sometimes be difficult due to their similarity to various other entities. A 10-year-old female reported swelling on the right side of the upper jaw for a month which was associated with mild continuous pain. On examination, a mild diffused swelling was noted on the right middle third of the face region which was firm in consistency and slightly tender. Intraorally, a firm tender swelling was noted on the right side of the hard palate. A proximal caries was noted with 55. A provisional diagnosis of dentoalveolar abscess with 55 was made. A panoramic radiograph showed loss of lamina dura concerning 11, 12, 53, 14, and 55, and loss of floor of the maxillary sinus. Cone-beam computed tomography and computed tomography-paranasal sinus revealed an ill-defined, hypodense osteolytic lesion with irregular borders extending from the 11 to 15 tooth region. Radiographic evaluation was suggestive of an infectious or neoplastic lesion. An incisional biopsy was performed and sent for histopathological and immunohistochemical analysis. A diagnosis of T-cell lymphoblastic lymphoma was made based on the features seen. The patient was sent for chemotherapy and radiotherapy. The reduction in the size of the lesion was noted on follow-up. Lymphoblastic lymphoma is a neoplasm of lymphocytes that is rarely seen in the oral cavity. Early diagnosis and prompt treatment are necessary to prevent further complications.

## Introduction

Lymphomas are malignant neoplasms of lymphocytes and their precursor cells. They can be broadly categorized as non-Hodgkin’s lymphoma (NHL) and Hodgkin’s lymphoma (HL) [1]. NHL represents about 90% of all lymphomas, the third most commonly affecting malignant disease in the oral tissues and bones of the jaw, after squamous cell carcinoma and malignant salivary gland tumors [2]. Approximately 75% of NHLs present as a nodal disease, and oral contribution is found only in 2% to 3% of patients. An aggressive form of NHL, lymphoblastic lymphoma (LBL), is seen in about 2% of NHL patients and is related to immature B lymphocytes (B-LBL) or T lymphocytes (T-LBL), with a yearly incidence of nearly 0.1-0.2 patients per 100,000 individuals in the United States [3-6].

Acute lymphoblastic leukemia (ALL) is the most commonly occurring malignant neoplasm in childhood. In the newest update of the World Health Organization categorization (2017) of hematopoietic tumors, LBL was classified as a single entity between lymphoma and T-cell lymphoblastic leukemia. It was differentiated entirely by the degree of engrossment existing in the bone marrow [7,8]. The diagnosis of LBL is made when there is below 25% permeation of lymphoblasts into the bone marrow [9].

T-LBL can show extranodal involvement in nearly 40% of the patients. However, its appearance in the oral cavity is usually uncommon, and there are few case reports in the literature [9,10].

Diagnosing the manifestations of oral lymphomas is challenging as they show clinical characteristics that mimic other diseases such as periodontitis, osteomyelitis, and other malignant disorders [11]. This might delay the accurate treatment, thus deteriorating the prognosis. Therefore, this case report aims to recognize the oral manifestations of lymphoma to assist oral health professionals in the initial diagnosis process.

## Case presentation

A 10-year-old female patient reported to the oral medicine and radiology department with a complaint of swelling over the right side of her upper jaw which was evident for one month. It had increased in size progressively up to the present size and was associated with mild, continuous, and non-radiating pain. The medical and family history were non-contributory. She gave no history of loss of weight, fever, wheezing, vomiting, nausea, or pain in the abdomen. No history of trauma could be elicited.

**Figure 1:**
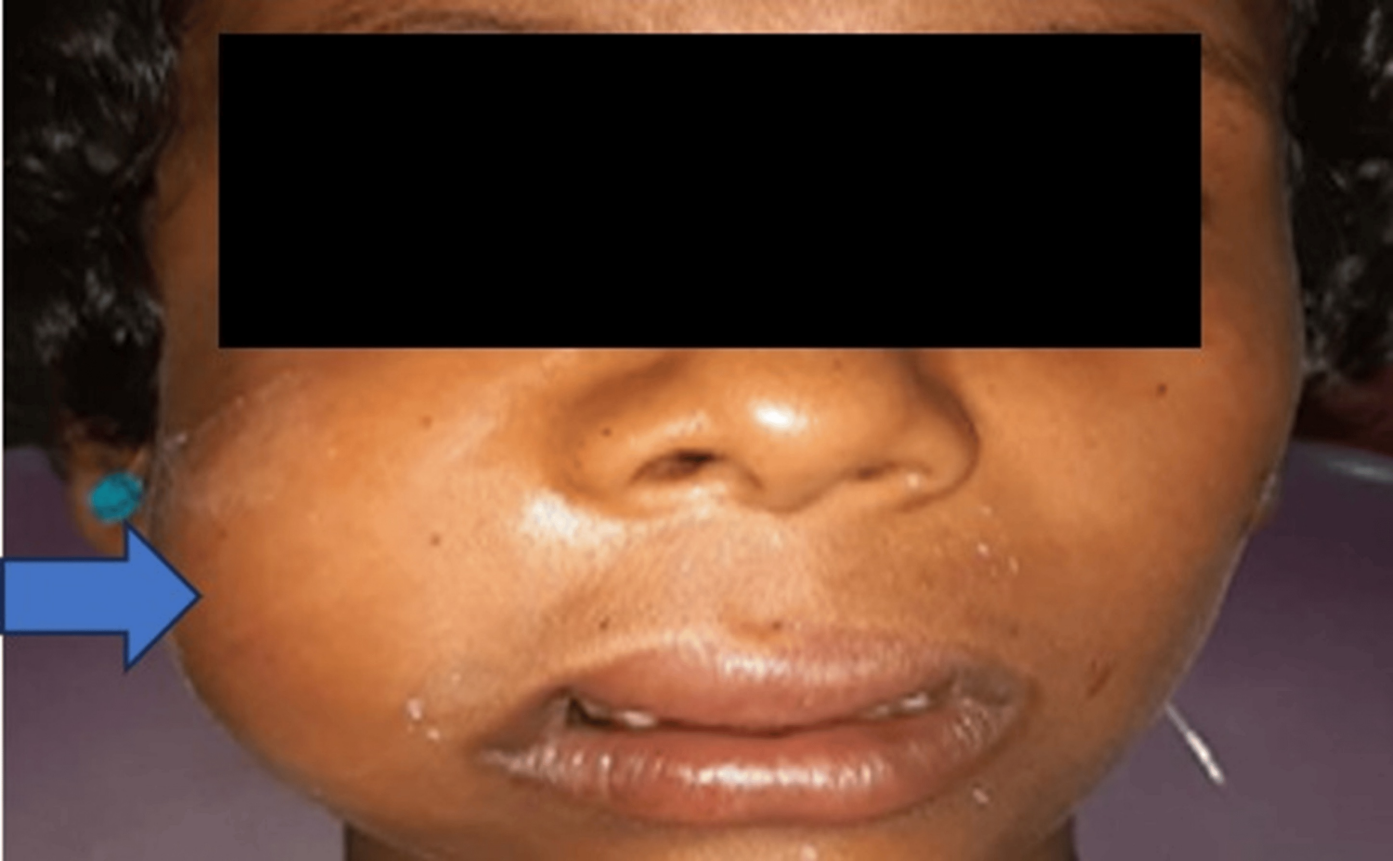
Frontal view of the patient showing severe diffuse swelling on the right middle third of the face.

On extraoral examination, gross facial asymmetry was noticed due to a solitary unilateral diffuse extraoral swelling over the right middle one-third of the face measuring approximately 4 cm × 3 cm in size (Figure 1). It extended anteriorly to posteriorly from the ala of the nose on the right side to 1 cm away from the Tagus of the right ear and from the infraorbital margin superiorly to the inferior border of the mandible on the right side inferiorly. The skin over the swelling appeared stretched and showed no color change. The swelling was firm in consistency with no local rise in temperature. There was mild tenderness on palpating the swelling. No clinical signs of lymphadenopathy were apparent.

**Figure 2:**
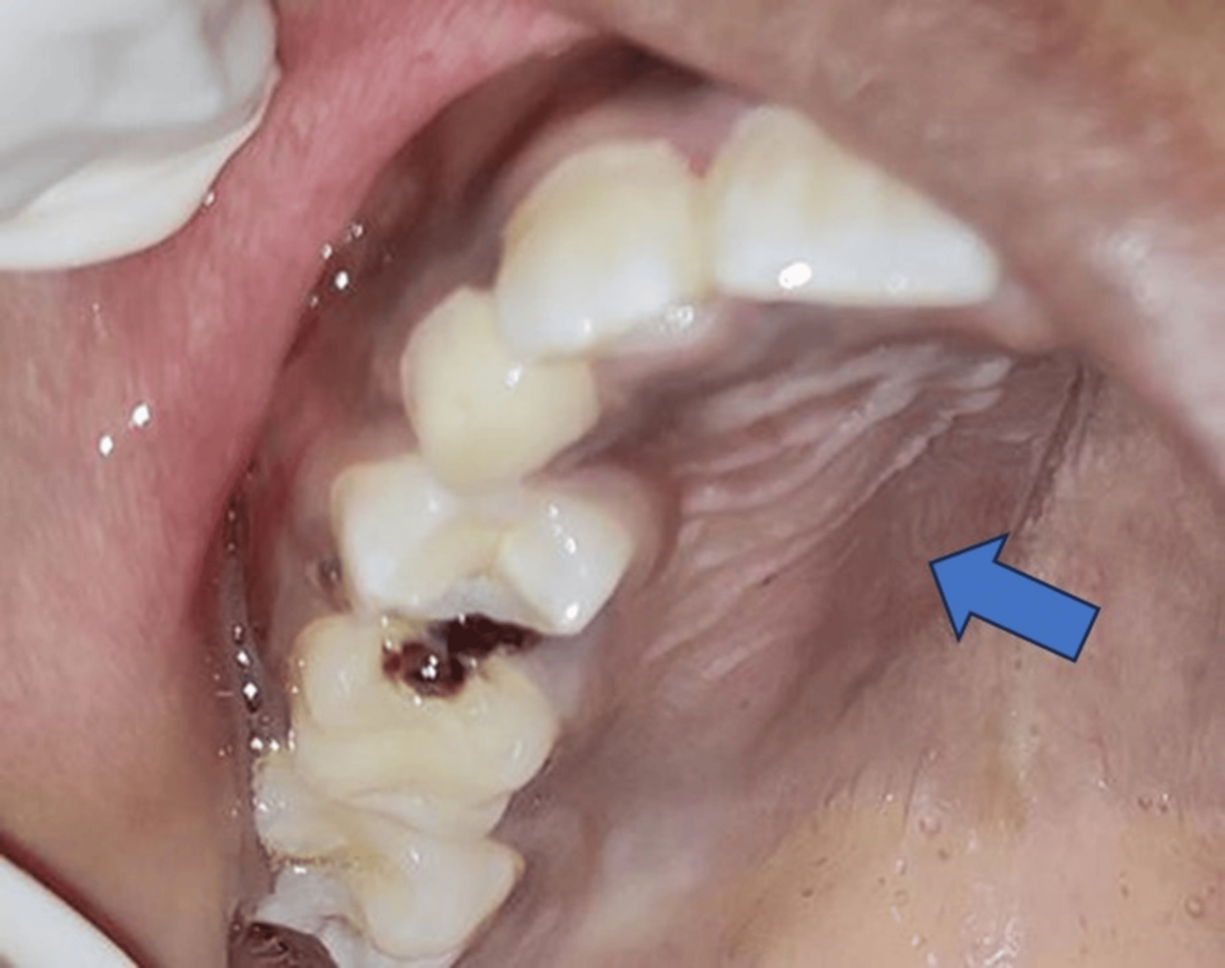
Intraoral view of the swelling seen on the hard palate.

On intraoral examination, a solitary well-defined swelling measuring approximately 5 cm × 4 cm was noticed on the right side of the hard palate which extended from tooth 11 to the distal aspect of tooth 55 anteroposteriorly and from buccal vestibule buccally to 3 cm palatal to the marginal gingiva of right upper teeth (Figure 2). The surface of the swelling was smooth and the overlying mucosa showed no color change. The consistency of swelling on palpation was firm and the occlusion was unaltered. Mild tenderness was elicited on palpation.

On hard tissue examination, a mixed dentition stage was seen. Proximal caries was seen with tooth 55. Grade 1 mobility was noted with teeth 11 and 16, and grade 2 mobility was noted with teeth 12 and 14.

Depending upon the history and clinical features, we made a provisional diagnosis of dentoalveolar abscess with 55, and a dentigerous cyst, benign fibro-osseous lesion, and benign odontogenic tumor were considered differential diagnoses.

**Figure 3:**
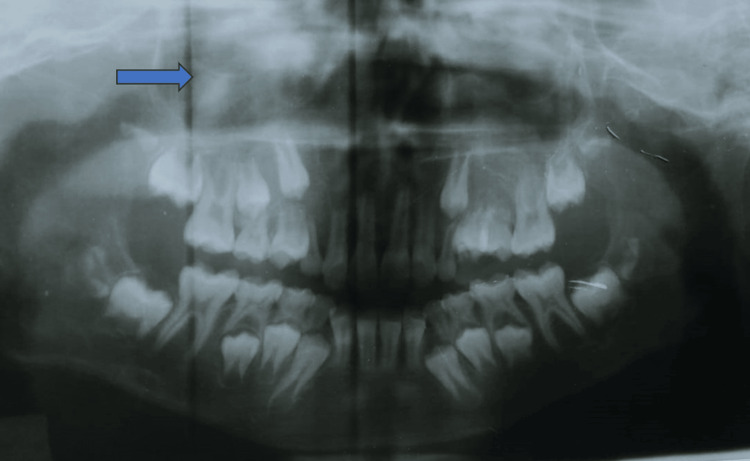
Panoramic image showing haziness and loss of floor in the right maxillary sinus.

**Figure 4:**
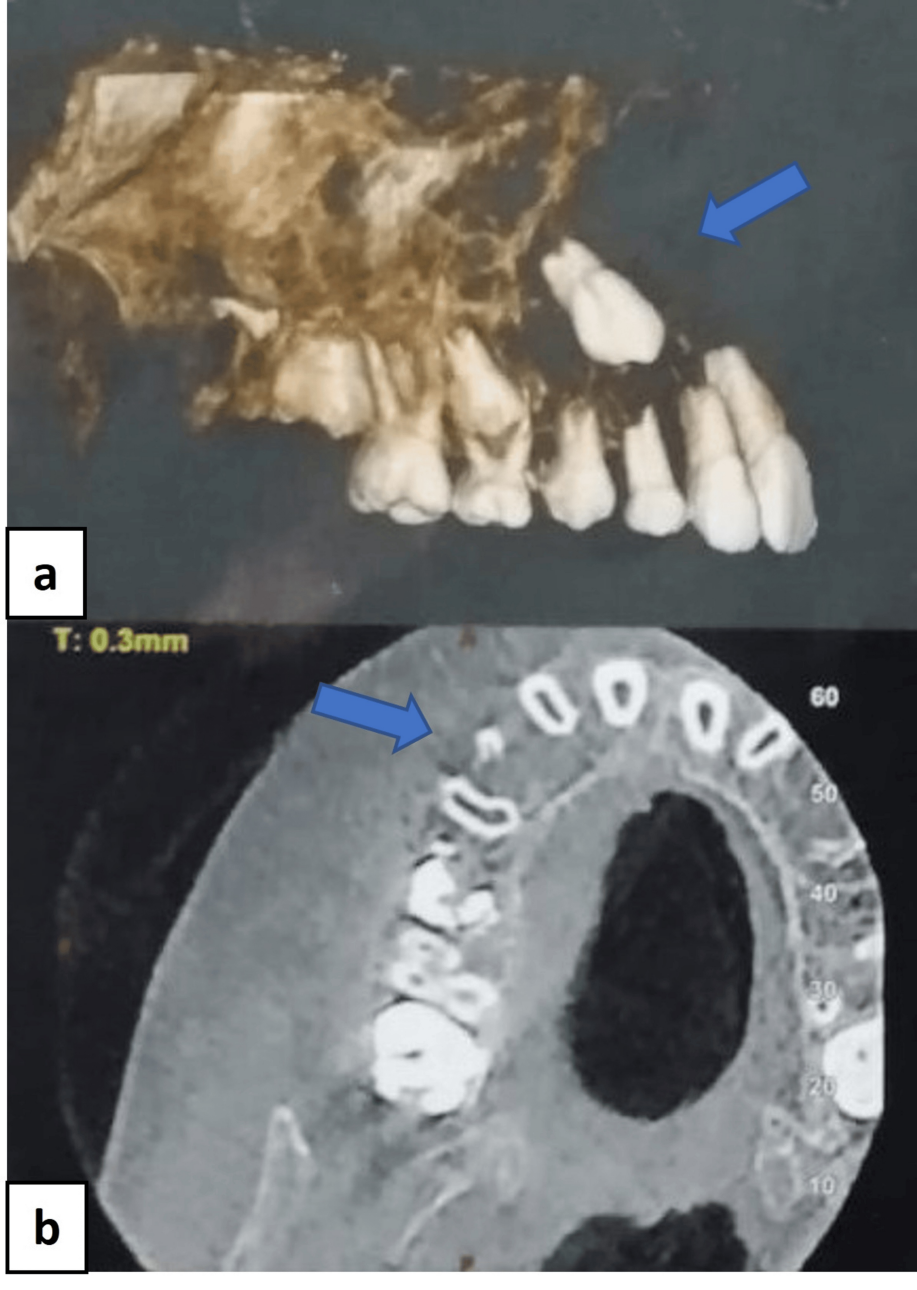
(a) Cone-beam computed tomography reconstructed image showing bone destruction in the right maxillary region. (b) Axial view showing an ill-defined, hypodense osteolytic lesion with irregular borders and periphery.

**Figure 5:**
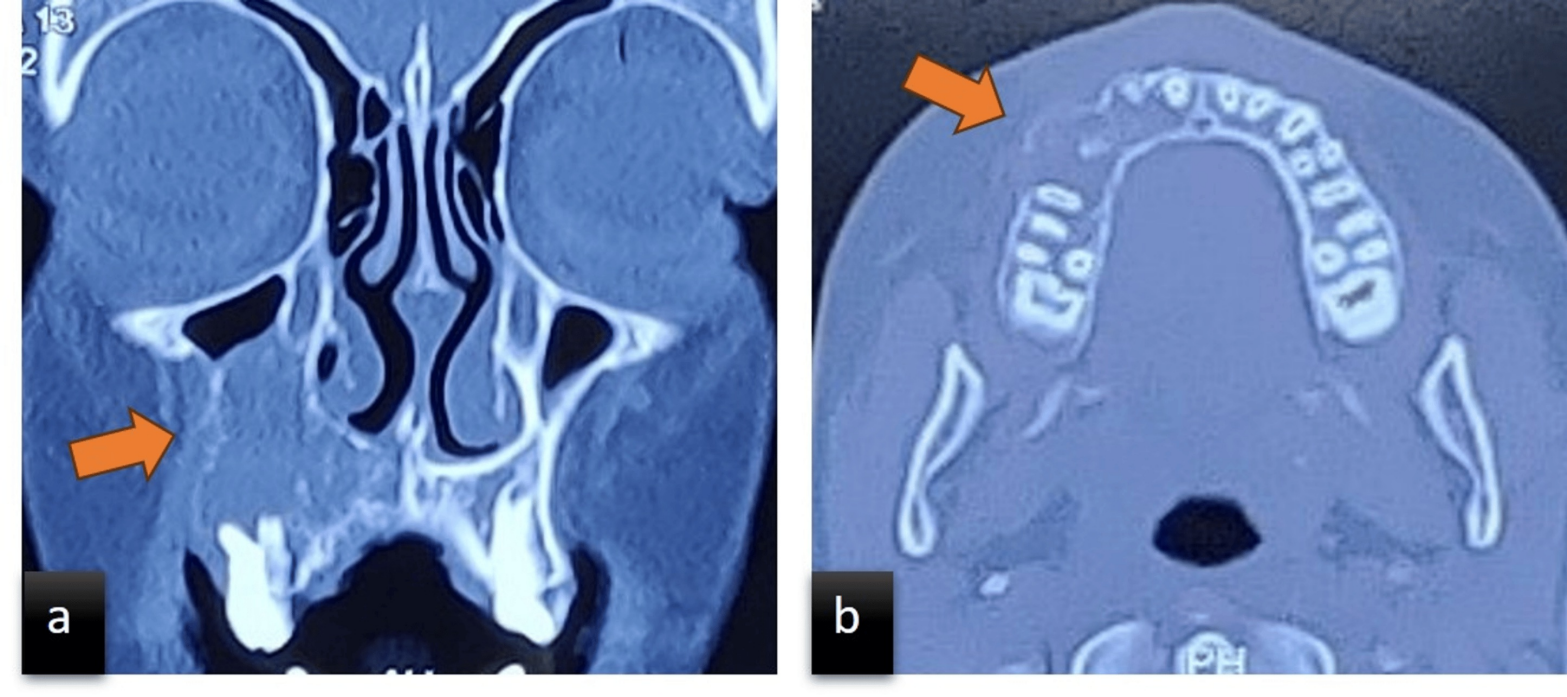
CT images showing (a) coronal section with an ill-defined hypodensity noted in the right maxillary region and invasion into the maxillary sinus. (b) Axial section with hypodensity in the right maxilla.

The patient was subjected to a panoramic radiograph which showed an ill-defined radiolucency on the right maxilla; erupting 15; retained deciduous teeth 53 and 55; loss of lamina dura with 11, 12, 53, 14, and 55; haziness of the right maxillary sinus; and loss of the right maxillary sinus floor (Figure 3). To evaluate the overall boundaries and invasion of the lesion into the surrounding regions, cone-beam computed tomography (CBCT) and computed tomography-paranasal sinus (CT-PNS) scans were advised. CBCT of the maxilla revealed an ill-defined, hypodense osteolytic lesion with irregular borders and periphery on the right maxilla involving the alveolar process of 11-15 regions, expansion of the buccal and palatal cortical plates with thinning and disruption of the cortical plates, and erosion of the floor and medial wall of the right maxillary sinus (Figure 4). The CT scan revealed irregular expansive soft tissue containing osteolytic lesion involving the right side of the maxilla involving the alveolar process of regions 11 to 15 with expansion, cortical thinning, and cortical defects. The soft tissue component was extending into the floor of the right maxillary sinus (Figure 5). After radiographic evaluation, the lesion was suggestive of a neoplastic or infective lesion.

**Figure 6:**
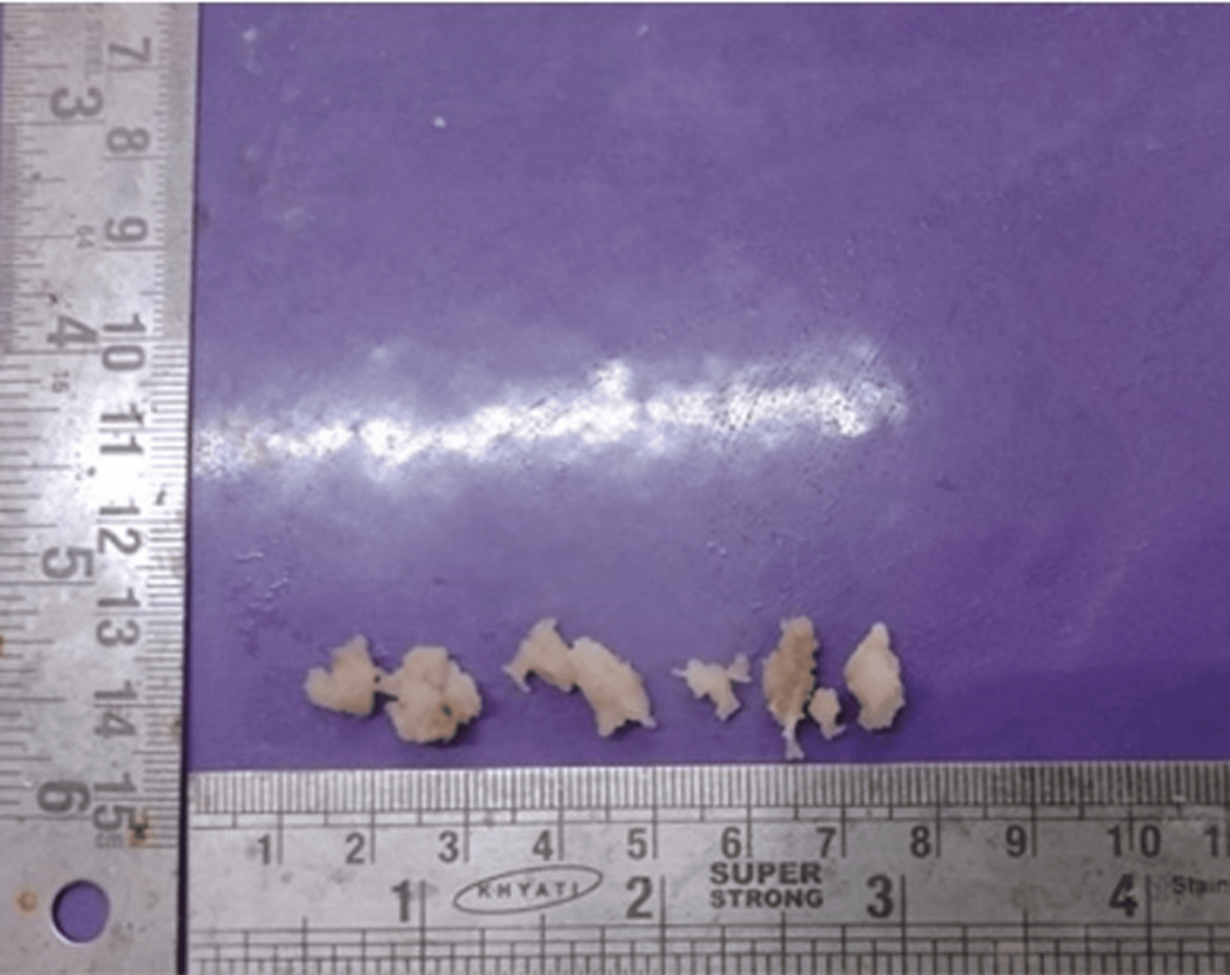
Creamish white soft tissue bits altogether measuring about 7 cm × 1.5 cm.

**Figure 7:**
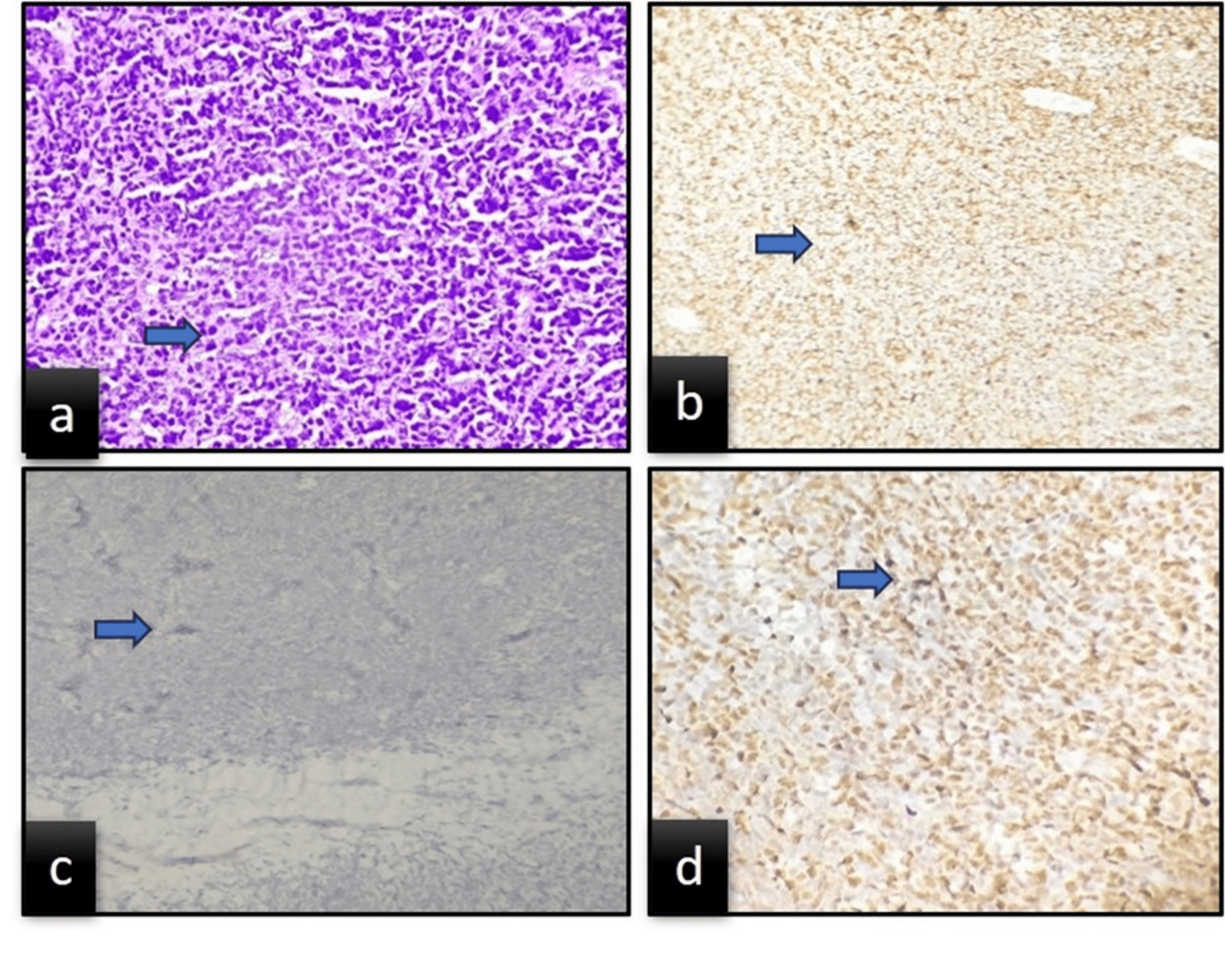
Histopathological and immunohistochemical examination. (a) Hematoxylin and eosin stain (40×) showing highly cellular stroma and sheets of small-to-intermediate-sized lymphoid cells. (b) Lymphoblast showing positivity for CD99. (c) No staining of cells by CD45 marker. (d) Positive staining of lymphoblast by TdT marker.

Before taking the incisional biopsy of the intraoral mass concerning the 55 region (Figure 5), various investigations were performed, including complete blood cell count, urine analysis, and calcium and alkaline phosphatase levels to rule out various diseases. The alkaline phosphatase levels were increased, and lymphocytosis was noted; the rest of the indices were within normal limits. The biopsy revealed sheets of round blue cells with minimal intervening connective tissue stroma and atypical features. Hence, a histopathological diagnosis was made as a malignant round-cell tumor. Immunohistochemistry was performed on the biopsy sample taken from the maxillary jaw lesion (Figure 6). Immunohistochemical analysis was positive for CD99 and TdT and negative for CD45, which was in favor of a rare diagnosis of T-LBL (Figure 7).

The patient was referred to the pediatric oncology department for full body evaluation. CT of the chest revealed two tiny nodular lesions in the right middle lobe. No malignant cells were present in the cerebrospinal fluid cytology. On performing bone marrow biopsy, marrow showed 80% cellularity with prominence of blasts. Bone marrow biopsy correlation with marrow cytology was consistent with ALL. Immunohistochemistry performed on the trephine biopsy specimen showed positive results for TdT, confirming the diagnosis of T-ALL/T-LBL. According to Murphy’s classification system, it was classified as stage IV [11].

**Figure 8:**
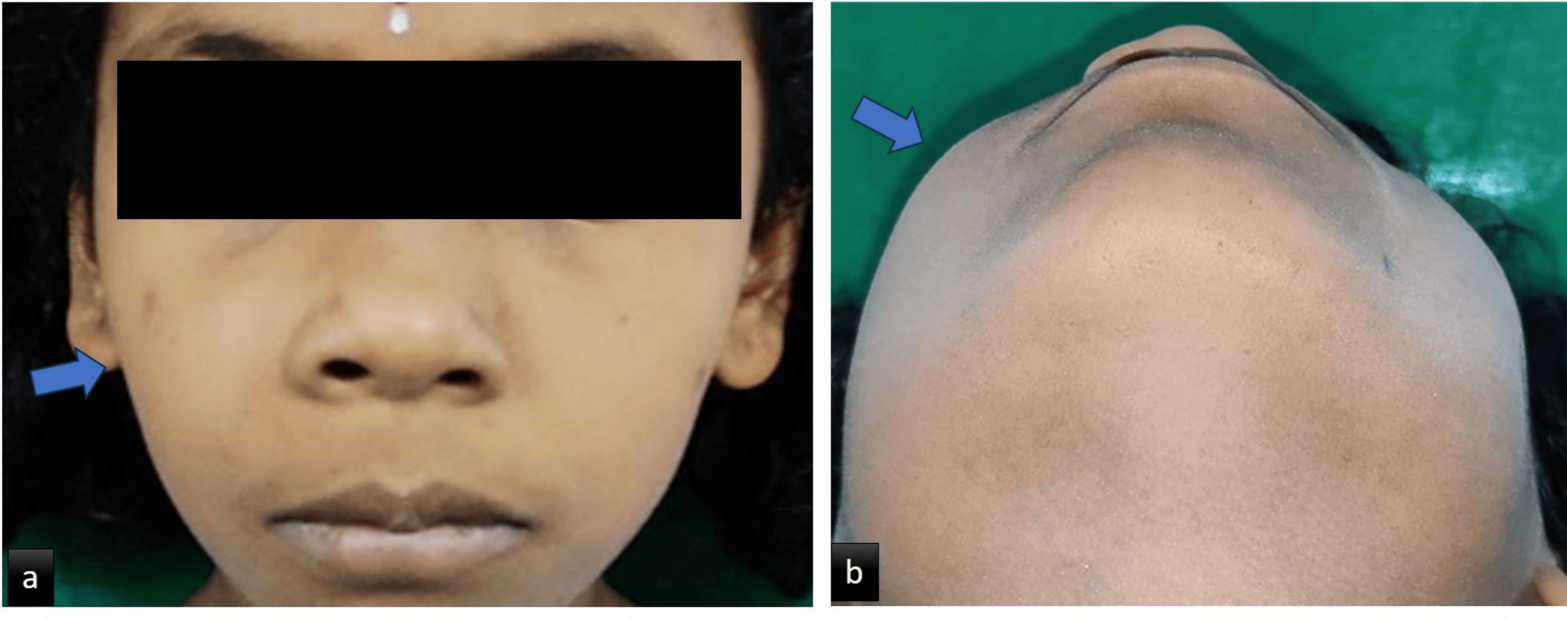
Follow-up after six months. (a) Frontal view of the patient showing regression in size of the lesion. (b) Axial view of the patient showing regression of the lesion.

After diagnosis, the patient underwent chemotherapy with tablet Wysolone 60 mg/m^2^ (D1-D29), injection vincristine 1.5 mg/m^2^ (D8, 15, 22, 29), injection Daunotec 30 mg/m^2^ (D8, 15, 22, 29), injection Leunase 5,000 U/m^2^ (D9, 12, 16, 19, 23, 26), intrathecal methotrexate 12 mg/dose (D8, 15, 29), followed by radiotherapy. On follow-up, a favorable result was observed with regression of the lesion present in the oral cavity (Figure 8).

## Discussion

Oral manifestations of lymphomas are seen in only 3% of all lymphoma patients and 4% of lymphoma patients with acquired immune deficiency syndrome (AIDS). It imitates a large family of oral pathologies such as periodontitis, pericoronitis, osteomyelitis, lockjaw, paresthesia, swelling, face asymmetry, and different malignancies of the oral tissues [12,13]. Nearly 40.52% of all lymphoma patients with intraoral findings are primarily incorrectly diagnosed and are treated by extractions or with periodontal or antibiotic therapy [12].

The causes of lymphoblastic lymphoma are not known. However, a likelihood of the connotation between exposure to a few viruses has been demonstrated, such as the human T-cell leukemia virus-1 (HTLV- 1) and Epstein-Barr virus (EBV) with peripheral T-cell lymphoma (PTCL) [14]. PTCL usually arises in patients with systemic illnesses such as AIDS, Sjogren’s syndrome, Crohn’s disease, rheumatoid arthritis, congenital immune deficiency, and celiac disease [15]. Our patient was not suffering from any of such illnesses and all the tests were non-productive for HIV antibodies, HTLV-1 antibodies, EBV, viral capsid antigen-IgM, and IgG.

Clinically, T-LBL is usually seen in male patients and presents with a huge mediastinal tumor and symptoms of respiratory distress. Additionally, skin, bones, gonadal, or central nervous system (CNS) can also be involved at times [16]. However, in the present case, there was no mediastinal connection. The five-year survival for stage I disease at diagnosis is 83.5% (25% of all diagnoses), while the survival for stage IV disease is 63.3% (33% of diagnoses) [5].

Sai et al. in 2012 reported a case of B-LBL in a 10-year-old female patient. The patient had an asymptomatic swelling in the mandibular region. The immunohistochemistry was positive for CD79 and TdT and negative for CD3 [17]. Our patient presented with a symptomatic swelling of the maxillary region. The immunohistochemistry was positive for CD99 and TdT and negative for CD45.

The panoramic radiograph of the current patient primarily showed no radiolucency. However, a more vigilant observation noted haziness in the right maxilla. These observations may not be perceptible to the untrained eye. Similarly, in a case by Goutzanis et al., the orthopantomagram revealed an ill-defined radiolucency in the ascending ramus [18].

Clinical stage evaluation of T-LBL includes a CT scan of the chest and abdominal-pelvis regions, evaluation of the bone marrow, and lumbar puncture. Accurate diagnosis of bone marrow’s tiny round cell tumors and various other neoplasms along with small-cell osteosarcoma, Ewing’s sarcoma, lymphoma, rhabdomyosarcoma, neuroblastoma, and neuroendocrine carcinoma is challenging because of the overlapping histology and comparable clinical and radiologic features. Lymphomas can be categorized by hematoxylin-eosin staining of tumor sections. However, immune phenotyping is usually the chief diagnostic technique [19].

Although the T-LBL and ALL characterize the same range of diseases, the chief manifestations are hardly seen in the oral tissues. They are randomly separated by the amount of involvement of the bone marrow. More than 25% of lymphoblasts containing cases in their bone marrow are known to have T-ALL. In our patient, oral swelling was the first reported symptom, after which, on whole body evaluation, 80% cellularity with prominence of blasts in the bone marrow was found. This case report is one of the few cases of T-ALL/LBL with oral manifestations.

There are different treatment choices for T-ALL/LBL such as chemotherapy, radiotherapy, or its amalgamation. Chemotherapy with CHOP (cyclophosphamide, doxorubicin, vincristine, and prednisone) is usually the first line of management [15]. According to the Murphy classification, T-LBL is classified in children [11]. Presently, CNS prophylaxis and intensive multidrug leukemia chemotherapy protocols are the standard therapy for LBL. They are based upon regimes with 7 to 10 drugs, such as prednisone, methotrexate, cyclophosphamide, vincristine, Elspar, cytarabine, etoposide, nitrosoureas, thioguanine, and anthracyclines, on a type-C base [20]. In the present study, methylprednisolone, vincristine, methotrexate, anthracycline, and asparaginase were used.

## Conclusions

Our case presented with no known cause of tooth mobility and with non-significant conventional radiographs. Advising appropriate investigations such as CBCT and CT led us to a proper diagnosis. The diagnostician must remember that a careful understanding of conventional radiographs might show evidence that implies malignancy which is generally ignored during routine radiographic interpretation. As the patient had no relevant medical history, consideration of LBL/lymphoma as a secondary differential diagnosis in any case of palatal swelling is necessary for advising appropriate investigations leading to the correct diagnosis, as the initiation of proper treatment at the earliest is crucial for patient survival and a good prognosis.
